# Space–time clusters and co-occurrence of *Plasmodium vivax* and *Plasmodium falciparum* malaria in West Bengal, India

**DOI:** 10.1186/s12936-024-05015-9

**Published:** 2024-06-17

**Authors:** Meghna Maiti, Utpal Roy

**Affiliations:** Department of Geography, University of Calcutta, Kolkata, 700019 India

**Keywords:** Vector borne diseases, Malaria, STL, Space–time scan statistics, Co-occurrence

## Abstract

**Background:**

Malaria, a prominent vector borne disease causing over a million annual cases worldwide, predominantly affects vulnerable populations in the least developed regions. Despite their preventable and treatable nature, malaria remains a global public health concern. In the last decade, India has faced a significant decline in malaria morbidity and mortality. As India pledged to eliminate malaria by 2030, this study examined a decade of surveillance data to uncover space–time clustering and seasonal trends of *Plasmodium vivax* and *Plasmodium falciparum* malaria cases in West Bengal.

**Methods:**

Seasonal and trend decomposition using Loess (STL) was applied to detect seasonal trend and anomaly of the time series. Univariate and multivariate space–time cluster analysis of both malaria cases were performed at block level using Kulldorff’s space–time scan statistics from April 2011 to March 2021 to detect statistically significant space–time clusters.

**Results:**

From the time series decomposition, a clear seasonal pattern is visible for both malaria cases. Statistical analysis indicated considerable high-risk *P. vivax* clusters, particularly in the northern, central, and lower Gangetic areas. Whereas, *P. falciparum* was concentrated in the western region with a significant recent transmission towards the lower Gangetic plain. From the multivariate space–time scan statistics, the co-occurrence of both cases were detected with four significant clusters, which signifies the regions experiencing a greater burden of malaria cases.

**Conclusions:**

Seasonal trends from the time series decomposition analysis show a gradual decline for both *P. vivax* and *P. falciparum* cases in West Bengal. The space–time scan statistics identified high-risk blocks for *P. vivax* and *P. falciparum* malaria and its co-occurrence. Both malaria types exhibit significant spatiotemporal variations over the study area. Identifying emerging high-risk areas of *P. falciparum* malaria over the Gangetic belt indicates the need for more research for its spatial shifting. Addressing the drivers of malaria transmission in these diverse clusters demands regional cooperation and strategic strategies, crucial steps towards overcoming the final obstacles in malaria eradication.

## Background

Worldwide, mosquito-borne diseases have led to a global societal impact, causing loss of life and necessitating substantial financial resources to maintain social stability. Several species of mosquitoes, which are the most frequent carriers of diseases, can cause dengue, chikungunya, malaria, and other illnesses [1]. Among them, malaria is a potentially fatal infectious disease that severely affects vulnerable communities in tropical and subtropical locations, where the environment is conducive to transmission. In 2021, the global estimate for malaria cases inflated to 247 million, marking an increase from the 230 million cases reported in 2015 [2]. Although, in 2022, an estimated 249 million malaria cases were reported, reflecting a 5 million case increase compared to the previous year [3]. Although, malaria deaths declined steadily from 864,000 in 2000 to 576,000 in 2019. In 2020, malaria deaths increased by 10% compared with 2019, to an estimated 631,000 and declined in 2022 to 608,000 [3]. Over the last decade, India has made significant progress in reducing malaria cases and deaths. In 2022, there were an estimated 176,522 reported cases, indicating a 14,769 increase compared to the previous year and the number of deaths has dropped significantly from 1018 in 2010 to 83 in 2022 [3]. The World Health Organization (WHO) Global Technical Strategy (GTS) for malaria fixed its target to eliminate malaria globally by 2030 Asia–Pacific countries, including India, have pledged to eliminate malaria by 2030 and reducing 50% mortality rate is a mandatory goal at the global scale [2]. The WHO GTS, the Asia Pacific Leaders Malaria Alliance (APLMA), the Malaria Elimination Roadmap, and the National Framework for Malaria Elimination (NFME) 2016–2030 have been developed together with partners and key stakeholders in a vision to eliminate malaria throughout the country by 2030 [4]. There is also a target to reduce the annual parasite index (API) to less than 1 by 2024 and contribute to improved health, quality of life and alleviating poverty [4]. To sustain zero indigenous morbidity and mortality, newer intervention tools were implemented, including early case detection and prompt treatment (EPDT) strategy, providing insecticide-treated bed nets (ITN) and long-lasting insecticidal nets (LLINs) to the residents for vector control, early diagnosis and prompt treatment with artemisinin-based combination therapy (ACT), and using indoor residual sprays (IRS) to protect at-risk population under integrated vector management (IVM) process [4].

Indian states such as Madhya Pradesh, Andhra Pradesh, Maharashtra, Bihar, West Bengal, Odisha and North East regions are highly prone to malaria endemicity, contributing around 97% of total malaria cases [5]. In the last decade, India has made tremendous progress in reducing malaria mortality and morbidity. Despite the steep decrease in malaria incidence across India, there are few endemic pockets where malaria remains a significant public health challenge to pose a stiff challenge to India’s malaria elimination efforts. In order to eliminate the parasite and prevent its recurrence, finding these final pockets of transmission is essential [6]. In the Indian state of West Bengal, malaria is predominantly caused by *P. vivax* and is also co-endemic with *P. falciparum*, considered the deadliest form of malaria, varied across due to different physiographic zones, political border (interstate and international) with high-endemic malaria region. Under the NFME, West Bengal is situated in the pre-elimination phase of Category 2 with an API of less than one and one or more districts reporting an API of more than one [4]. In 2018, the Health and Family Welfare Department of the Government of West Bengal officially declared malaria as a notifiable disease to entail on-time diagnosis and reporting by all government and private hospitals/laboratories, including non-governmental organization (NGO) run hospitals, as well as individual medical practitioners to strengthen capturing of surveillance data which is a matter concern [7].

Climatic sensitive malaria fever exhibits a seasonal variation in India where incidence of cases typically increases during the monsoon season, which lasts from June to September [8, 9]. The high humidity and rainfall during this season create favourable breeding conditions for the *Anopheles* mosquito, which is the primary vector for transmitting the malaria parasite [8]. The incidence of malaria cases typically peaks in August and September, and declines towards the end of the year. Understanding the seasonal trend and anomaly in malaria cases can lead to identify the underlying factors that drives the pattern which is crucial for planning and implementing effective prevention and control measures [10]. Analysing the seasonal trend in malaria cases can enable to proactively handle potential outbreaks or spikes in malaria cases and make well-informed decisions [6, 11]. Additionally, knowledge about the seasonal pattern of malaria transmission can assist in estimating the transmission period and implementing appropriate and effective control measures [12]. A positive approach towards understanding the seasonal variation in malaria cases can significantly reduce the incidence of malaria and prevent malaria-related fatalities. Space–time disease mapping of malaria outbreaks is an informative tool for public health interventions that provide information like rate of transmission, cyclical pattern, intensity and risk of diffusion to the new location, persistent nature [13, 14]. Malaria cluster identification can aid in the demarcation of problem regions and the deployment of focused programme interventions suited for the eradication phase [6]. Focused interventions in malaria-risk regions are expected to be more cost-effective than uniform resource allocation, especially in resource-constrained settings for long-term eradication programmes [15–17].

Scan statistics is one of the most common statistical methods used to identify the clusters of cases spatially and temporally [18]. Whereas Kulldorff’s univariate (STSS) identifies space–time clusters of single diseases, multivariate STSS can evaluate clusters of multiple diseases that co-occurred [19, 20]. Univariate space–time statistics have been used to identify the outbreaks and space–time clusters of diseases, such as malaria [6, 21, 22], dengue [23] and chikungunya [24], COVID-19 [25], lyme disease [26]. Space–time scan statistics were also used to identify the cluster pattern of *P. vivax* and *P. falciparum* individually in Bhutan [6], Ahmedabad City [27], Karnataka [28]. Whereas multivariate space–time scan statistics can examine space–time clusters of the simultaneous co-occurrence or co-existence of multiple diseases at one point of time used to identify outbreaks of dengue and chikungunya in Colombia [23].

It is important to consider that the use of 2D techniques [6, 25, 26, 28] to visualize space time clusters may not always provide a complete representation of space–time patterns of clusters. However, there are some remarkable examples in the literature where the incorporation of 3D visualization has proven to be effective in showcasing the temporal variation of clusters that could be missed through 2D techniques. Present study emphasizes the importance of incorporating 3D visualization techniques to provide a more comprehensive understanding of the space–time patterns of clusters which comprises the analysis of fluctuations in size and duration, the movements of clusters over time, the simultaneous occurrence of *P. vivax* and *P. falciparum*, and the reappearance of significant clusters.

Above all it is crucial to comprehend the spatio-temporal distribution of malaria cases at the block level for developing intervention strategies since West Bengal has a porous international border with Bangladesh and a national border with high endemic states. However, no large-scale studies have examined geographic patterns of both *P. vivax* and *P. falciparum* malaria in West Bengal. This study is the first attempt to evaluate seasonal variability and the retrospective space–time distribution of individual and co-occurring—*P. vivax* and *P. falciparum* malaria across all 341 blocks in West Bengal. The purpose of the present study is to fill this gap in the understanding of seasonal patterns and the spatial and spatiotemporal distribution of two types of malaria and to estimate the relative risk of each high space time cluster at block level in West Bengal during 2011–2021.

## Methods

### Study area

West Bengal is located in the eastern part of India between latitudes of 21° 31′–27° 14′ N and longitudes of 85° 49′–89° 51′E, sharing an international border with Bangladesh in the east, Bhutan and Nepal in the north. It also shares interstate boundaries with Sikkim, Assam, Jharkhand, Odisha and Bihar. It is the fourth most populous state, which occupies 88,752 sq. km (34,263 sq. miles) with 91 million inhabitants. This Indian state has a significant variation in climate due to its geographical diversity. The hot and dry seasons belong to the western part, and wet and cold in the northern part, including warmer conditions in the coastal areas in the southern part, are the most significant. Belonging to a monsoon climatic region, Bengal faces summer, winter and rainy seasons most significantly.

### Data

As malaria is a vector-borne notifiable disease, the number of positive test results have to be reported by the laboratories to the Department of Health, West Bengal, through the National Vector Borne Disease Control Programme (NVBDCP). In West Bengal, routine diagnosis of malaria is performed by rapid diagnostic test (RDT), as NVBDCP recommends, and gold microscopy is used more for confirmatory diagnosis. Malaria surveillance is carried out through the public health sub-centre, District Hospitals (DH), Block Primary Health Centre (BPHC), Primary Health Centres (PHC) and Rural Hospitals (RH). Slides are collected by Accredited Social Health Activists (ASHA), Auxiliary Nursing Midwifery (ANM) and other health workers in the community in all blocks. After slide examination through the respective diagnosis unit, all positive test reports will be submitted to the Department of Health for further analysis. A monthly report on malaria is published by the Health Management Information System (HMIS) (https://hmis.mohfw.gov.in/#!/), Ministry of Health and Family Welfare, Government of India. However, a dataset containing reported positive cases of two malaria species, *P. vivax* and *P. falciparum*, has been collected for subsequent analysis for each of 341 blocks (excluding Kolkata urban metropolitan city). During the specified time periods, the monthly data was effectively utilized to capture relevant events. The datasets were compiled, and the cases were geocoded to the block level with the help of ESRI Arc GIS 10.8.1. Relevant population-related data are taken from the last national Census (https://censusindia.gov.in/census.website/) for subsequent analysis.

### STL and anomaly detection

STL or ‘Seasonal and Trend decomposition using Loess’ is a versatile and robust method for decomposing time series developed by Robert Cleveland and others in 1990 which uses locally fitted regression models to decompose a time series into trend, seasonal and residual or remainder components [29]. With the help of two loops, the STL algorithm smoothes the time series using LOESS; the inner loop alternates between seasonal and trend smoothing, while the outer loop reduces the impact of outliers. At first, the seasonal component is calculated during the inner loop and then subtracted before the trend component is calculated. The residual is calculated by subtracting the seasonal and trend components from the time series, which indicate the amount of noise present in the data. Small values suggest accurate seasonal and trend components, while larger values indicate noise in the data. In this study, additive decomposition was used as follows:

$${Y}_{t}={T}_{t}+ {S}_{t}+{R}_{t}$$

where ${Y}_{t}$ represents the number of malaria cases with logarithmic transformation, ${T}_{t}$ is the trend component, ${S}_{t}$ is the seasonal component, ${R}_{t}$ is the residual component, for $t$ = 1 to N (month) time. In addition, from the residual component, anomaly detection was also incorporated which deviates significantly from the normal time series, helping to detect the extreme condition of monthly observation of malaria cases.

### Space time scan statistics

In this study, Kulldorff’s retrospective univariate and multivariate Space Time Scan Statistics (STSS) were applied in SaTScan™ software v.10.1 [18] to identify statistically significant space–time clusters and to estimate the relative risk (RR) of *P. vivax* and *P. falciparum* of malaria species on a successive 120-month period from April 2011 to March 2021. In order to identify space–time clusters of disease incidence, a cylindrical scanning window with a circular geographic base and a height that corresponds to time is placed over the research region and moves from one point to another. The age structure of the population may influence the incidence of disease, however, due to the inaccessibility of the age and sex data at this time for cases in this study, the assumption has been made that malaria incidence follows a Poisson distribution according to the block level population, e.g. the assumed population at risk. According to the null hypothesis, the model depicts an inhomogeneous Poisson process with an intensity that is proportionate to the population at risk. Whereas the alternative hypothesis is that there are more instances of malaria than would be predicted under the null model.

The base of each cylinder is centred on the centroid of a particular block of the study region and expanded in both the space and time direction until a maximum spatial and temporal threshold is reached. In this study it relaxes the restriction on the cylinder end point searching where the cylinder scans each point throughout the time period to get the cluster at any time. The maximum size of the spatial window was set at 25% of the exposed population and the temporal window of the space time cylinder was set at 25% of the study period with no geographical overlapping of clusters. Also, 999 Monte Carlo replications for the testing of statistical significance at p value < 0.05 was used to ensure adequate power for defining a cluster. Mapping of the significant clusters and relative risk of univariate and multivariate *P. vivax* and *P. falciparum* were done using the GIS ArcMap and ArcScene 10.8.1.

## Results

### Exploratory analysis

**Fig. 1:**
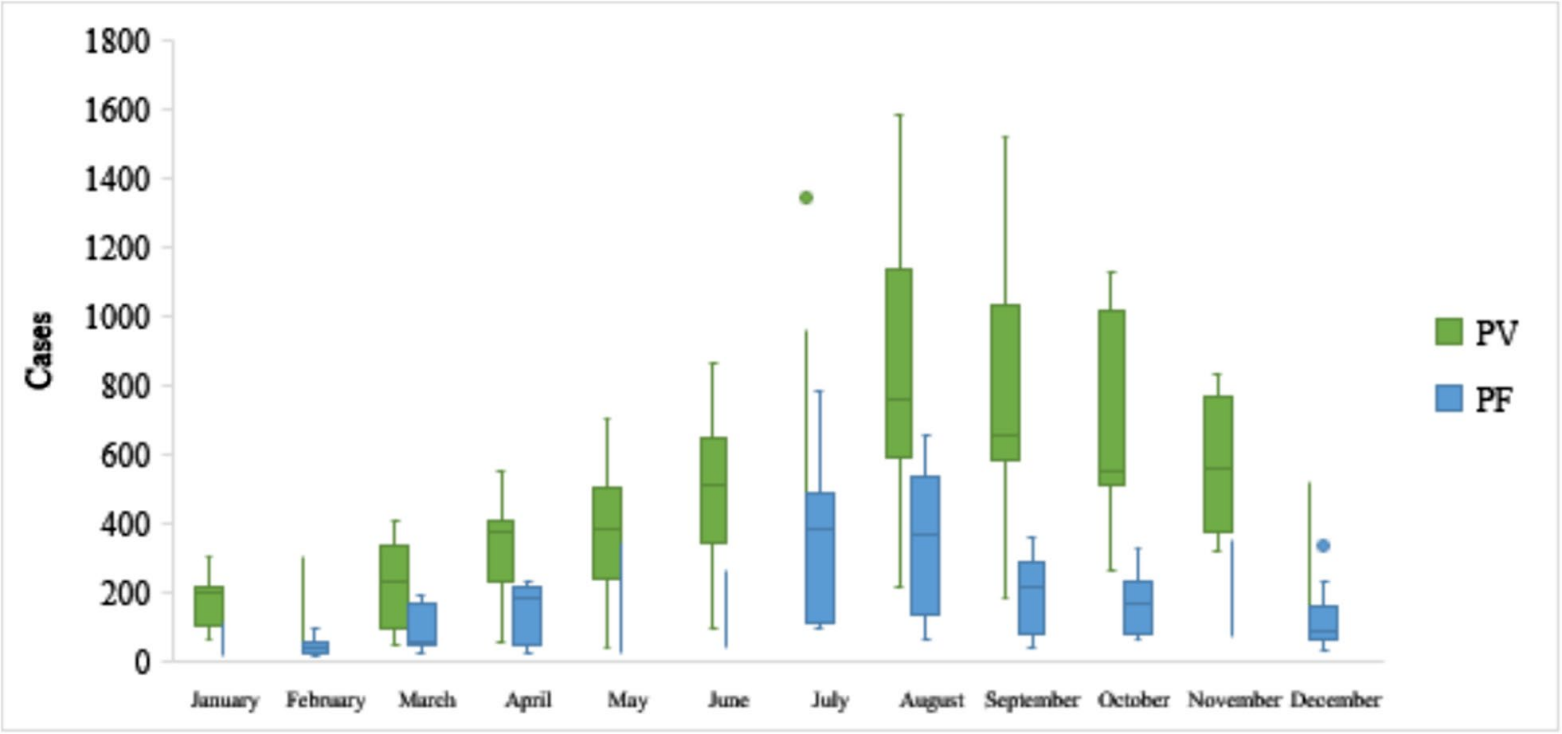
Monthly distribution of *P. vivax* and *P. falciparum* malaria in West Bengal during the study period

During the study period (April 2011 to March 2021), n = 55,476 *P. vivax* cases and n = 20,844 *P. falciparum* cases were positively reported across 341 Blocks in West Bengal. *Plasmodium vivax* accounted for 72.6% of malaria cases, while 27.3% were *P. falciparum*. Figure 1 displays the box plot with the monthly distribution of *P. vivax* and *P. falciparum* malaria cases across 341 Blocks in West Bengal during the study period, which represents the value of the interquartile range of 25th, 50th and 75th percentile and whiskers with dispersion. Though both types of malaria were observed throughout the year, they tended to increase from June to November, the monsoon and post-monsoon season in West Bengal. Throughout the study period, 71.15% of *P. vivax* cases were observed during monsoon and post-monsoon periods, while 70.24% were *P. falciparum*. Also, a substantial decrease in *P. vivax* and *P. falciparum* malaria cases was observed during the summer and winter seasons during the study period. The various seasons or climatic conditions play an important role in seasonal variations of malaria vector distribution. Maximum dispersion took place during the monsoon period.

### Time-series decomposition and anomaly detection

**Fig. 2:**
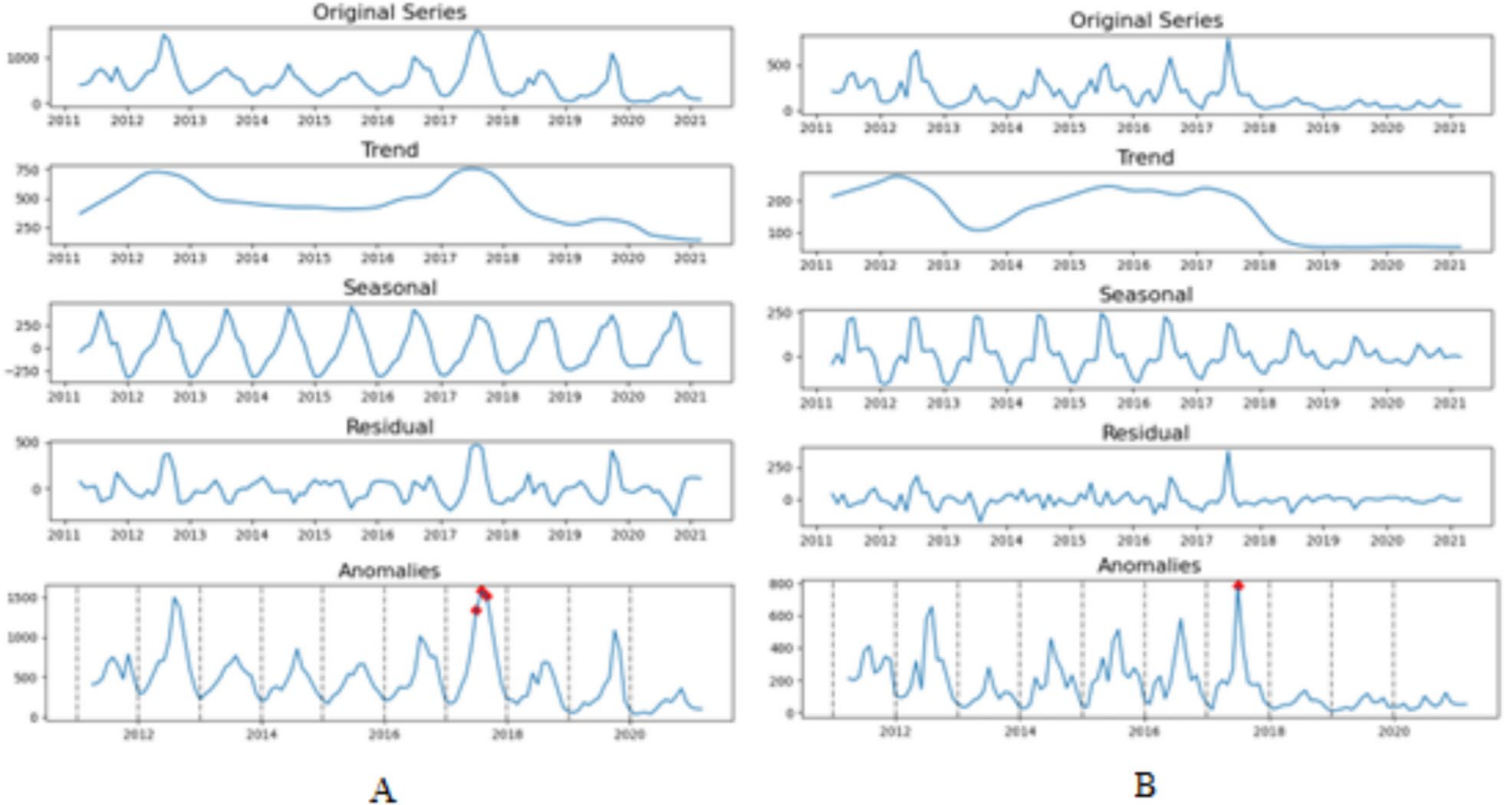
Decomposed time-series and anomaly detection of (**A**) *P. vivax* and (**B**) *P. falciparum* cases

From the time series decomposition, a clear seasonal pattern is visible for both *P. vivax* (Fig. 2A) and *P. falciparum* (2B). The seasonal component of STL highlighted the recurring temporal pattern (with the shape of an oscillating or wave pattern). For *P. vivax*, a high count occurred during the monsoon season of June and July months and a low count in January, with the oscillation decreasing narrowly over time indicating a slower rate of *P. vivax* occurrence. Whereas, in the case of *P. falciparum*, two small peaks are associated before and after the monsoon month of June and oscillation decreases in amplitude over time which indicates that the seasonal variation is decreasing over time. The inter-annual pattern showed two large peaks during mid-2012 and mid 2017 for *P. vivax*, and one large peak during early 2012 with a plateauing peak during early 2015 to late 2017 for *P. falciparum*, followed by a dropping nature afterwards. The residuals are also visible during the time frame for both of the cases with an oscillating pattern. The number of *P. vivax* cases are increased during monsoon and post monsoon seasons throughout the time frame. Whereas for *P. vivax* the residual values begin relatively high and become smaller after 2017. Anomalies were identified during July 2017 (n = 1339), August 2017 (n = 1583) and September 2017 (n = 1520) for *P. vivax* and during July 2017 (n = 783) for *P. falciparum* as an outlier located outside the threshold limit. However, the annual number of both malaria showed a considerable decrease especially after 2018.

#### Univariate space–time cluster of *P. vivax*

The univariate space–time cluster analysis of monthly *P. vivax* malaria cases identified a non-random distribution of cases at the Block level in West Bengal from April 2011 to March 2021. The analysis detected seven significant clusters affecting 157 blocks for *P. vivax* cases (Table 1). A column with no of blocks represents associated blocks that form a cluster. The most observed cases (n = 5068) were found in cluster 1, centred at the Binpur-II block, with the highest relative risk (RR = 42.95) from April 2011 to November 2014, located on the western side of the study area. Three clusters were detected in 2017 centred at Kalchini (cluster 2 from June to December 2017), Magrahat-II (cluster 4 from August to October 2017) and Mangolkote (cluster 7 from July to October 2017). Cluster 3, centred at Farakka with 34 blocks, was detected as the most extended temporal cluster from April 2012 to November 2016, with the highest observed cases (n = 9184). In 2018, one cluster was detected centred at Jaypur in cluster 5, and cluster 6, centred at Pandua from June 2011 to August 2013, created a self-cluster (without any radius). Out of seven clusters, it is visible that clusters 1 and 3 have the most prolonged duration, claiming the persistent nature of *P. vivax*.

**Table 1:** Univariate pace-time cluster of *P. vivax*

Cluster	Time period	Observed cases	Expected Cases	No. of blocks	Relative risk (RR)	p value
1	April 2011–November 2014	5068	129.55	3	42.95	< 0.001
2	June 2017–December 2017	2025	81.67	7	25.69	< 0.001
3	April 2012–November 2016	9184	2945.30	34	3.54	< 0.001
4	August 2017–October 2017	1080	276.38	62	3.97	< 0.001
5	February 2018–November 2018	337	22.79	2	14.87	< 0.001
6	June 2011–August 2013	220	56.28	1	3.92	< 0.001
7	July 2017–October 2017	411	237.84	47	1.73	< 0.001

**Fig. 3 A:**
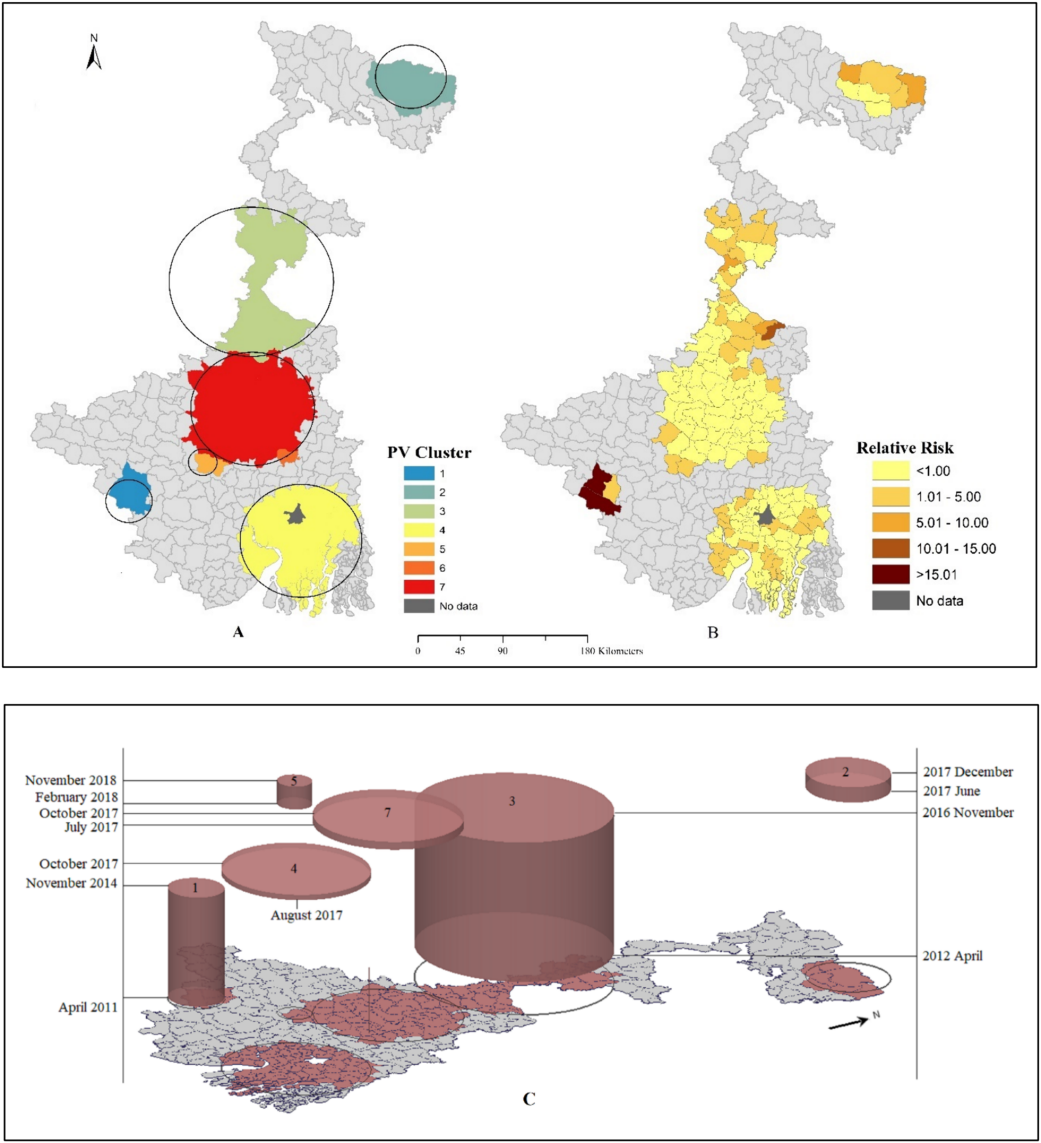
Univariate space–time cluster, and **B** Statistically significant relative risk of *P. vivax.* **C** 3D visualization of univariate space–time cluster of *P. vivax*

Figure 3A represents the space–time cluster of *P. vivax* cases, and throughout the study period, *P. vivax* cases varied across the study area. Right side Fig. 1B depicts each block's statistically significant relative risk of *P. vivax* from the space–time clusters. Out of 341 blocks, only two were assigned zero relative risk due to no observed cases reported. From 156 blocks assigned to a cluster, 102 blocks reported relative risk < 1 due to lower observed than expected cases. The rest of the 54 blocks have higher than one relative risk due to the higher observed than expected cases. In comparison, three blocks reported more than ten relative risks, including Bhagawangola-II (RR = 11.11) from cluster 3, Binpur-II (RR = 22.47) and Ranibadh (RR = 31.9) from cluster 1.

#### Univariate space–time cluster of *P. falciparum*

The univariate space–time cluster analysis of monthly *P. falciparum* malaria cases identified a non-random distribution of cases at the Block level in West Bengal from April 2011 to March 2021. The analysis detected 18 significant clusters affecting 69 blocks for *P. falciparum* cases (Table 2). The most observed cases (n = 8224) found in cluster 1, centred at Barabazar block from October 2011 to September 2016, have the most extended duration cluster. Out of 18 clusters, 10 clusters form a self-centred cluster due to higher observed cases. Eight clusters were detected from 2011 to 2016, centred at Matiali (cluster 2 from July 2011 to December 2011), Kaliachak-I (cluster 3 from August 2012 to November 2012), Illambazar (cluster 4 from June 2013 to July 2013), Khoyrasol (cluster 6 during July 2011 to September 2011), Murshidabad Jiaganj (cluster 7 during July 2012 to November 2012), Potashpur- I ( cluster 9 during June 2011 to November 2012), Kolaghat (cluster 11 during October 2013 to February 2016) and Tahatta (cluster 14 during November 2014 to March 2015). Two thousand seventeen two clusters were reported, centred at Manteswar (cluster 12 from April 2017 to May 2017) and Mandirbazar (cluster 16 from August 2017 to November 2017). From 2018 to 2019, two clusters were detected, centred at Hanskhali (cluster 13 from July 2018 to February 2019) and Shyampur (cluster 17 from September 2018 to Oct 2018). From 2020 onwards, five clusters were identified with a duration between one to three months, out of which three clusters centered at Jangipara (cluster 5 from January 2020 to March 2020), Haringhata (cluster 8 from February 2021), Baruipur (cluster 18 from October 2020 to November 2020). Another two clusters centred at Ranaghat-I (cluster 10) and Moyna (cluster 15) from January 2021 to March 2021. While the highest observed cases were reported from cluster 1, cluster 4 was identified with the highest relative risk due to the lowest number of expected *P. falciparum* cases.

**Table 2:** Univariate space–time cluster of *P. falciparum*

Cluster	Time period	Observed cases	Expected cases	No. of blocks	Relative risk (RR)	p value
1	October 2011–September 2016	8334	603.92	31	23.07	< 0.001
2	July 2011–December 2011	192	1.66	1	116.57	< 0.001
3	August 2012–November 2012	183	5.68	2	32.49	< 0.001
4	June 2013–July 2013	94	0.80	1	118.70	< 0.001
5	January 2020–March 2020	95	1.57	1	60.89	< 0.001
6	July 2011–September 2011	63	1.08	1	58.29	< 0.001
7	July 2012–November 2012	172	23.89	8	7.25	< 0.001
8	February 2021–February 2021	25	0.54	1	45.94	< 0.001
9	June 2011–November 2012	47	7.36	1	6.40	< 0.001
10	January 2021–March 2021	21	1.52	1	13.81	< 0.001
11	October 2013–February 2016	64	19.84	1	3.23	< 0.001
12	April 2017–May 2017	20	1.83	2	10.95	< 0.001
13	July 2018–February 2019	30	5.53	1	5.43	< 0.001
14	November 2014–May 2015	20	2.50	1	8.02	< 0.001
15	January 2021–March 2021	23	5.01	3	4.60	< 0.001
16	August 2017–November 2017	20	3.86	2	5.19	< 0.001
17	September 2018–October 2018	23	6.90	7	3.34	< 0.001
18	October 2020–November 2020	20	5.76	4	3.48	< 0.001

**Fig. 4 A:**
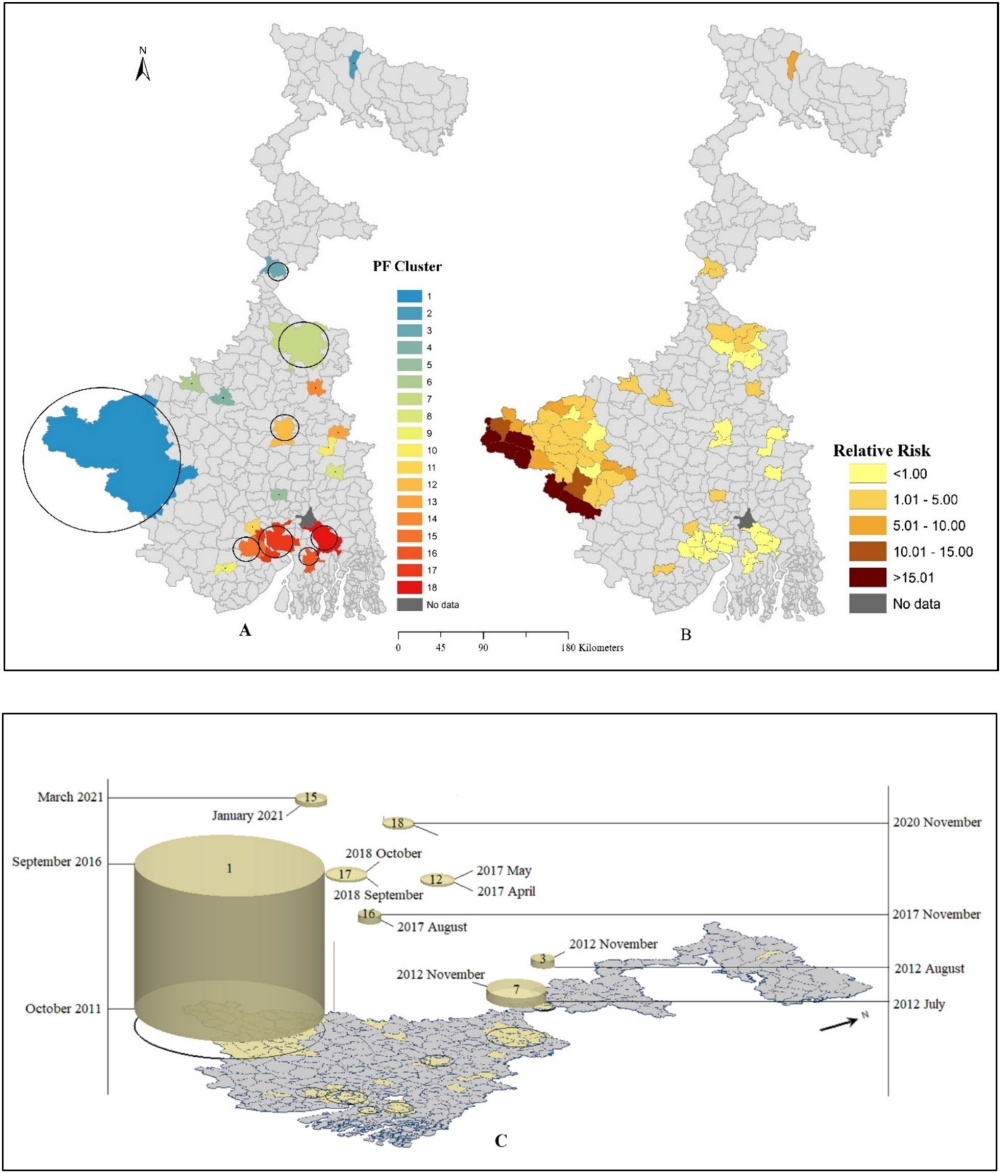
Univariate space–time cluster, and **B** Statistically significant relative risk of *P. falciparum.* **C** 3D visualization of univariate space–time cluster of *P. falciparum*

Figure 4A represents the space–time cluster of *P. falciparum* cases from April 2011 to March 2021. Right side Fig. 4B depicts each block's statistically significant relative risk of *P. falciparum* from the space–time clusters. Out of 341 blocks, 25 blocks assigned zero relative risk due to no observed *P. falciparum* cases reported. Of 69 blocks assigned to a cluster, 28 blocks reported relative risk < 1 due to lower observed than expected cases. The other 41 blocks have higher than one relative risk due to the higher observed than expected cases. From cluster 1, out of 31 blocks eight blocks reported relative risk > 10 including Ranibadh (RR = 10.89), Jhalda- II (RR = 14.04), Arsha (RR = 21.35), Binpur-II (RR = 27.76), Jhalda- I (RR = 28.97), Balaramapur (RR = 30.66), Bagmundi (63.35) and Banduan (RR = 80.02).

### Multivariate space–time cluster

Table 3 summarizes the result of the multivariate space–time clusters analysis, where nine clusters detected the coexistence of *P. vivax* and *P. falciparum*, affecting 133 blocks. Clusters 1 to 3 and 5 were formed with *P. vivax* and *P. falciparum*, while clusters 4 and 9 were formed with only *P. vivax* and clusters 6—8 were formed with *P. falciparum*. Four clusters detected with *P. vivax* and *P. falciparum* centred at Balarampur (cluster 1 from April 2011 to March 2016 with 16,276 observed cases), Farakka (cluster 2 from April 2012 to November 2016 with 10,539 observed cases), Kalchini (cluster 3 from June 2017 to December 2017) and Jaypur (cluster 5 during February 2018 to November 2018). While Cluster 4, centred at Magrahat- II (from August 2017 to October 2017) and Cluster 9, centred at Pandua (from June 2011 to August 2013), formed with *P. vivax* cases only, though *P. falciparum* cases were observed there, the analysis could not find any statistically significant space–time cluster. An alternative scenario took place with three clusters, including Cluster 6, centred at Illambazar (from June 2013 to July 2013); Cluster 7, centred at Jangipara (from January 2020 to March 2020); and Cluster 8, centred at Khoyrasol (from July 2011 to September 2011) with *P. falciparum* cases only. There was no evidence to construct a significant space–time cluster in those cases.

**Table 3:** Multivariate space–time cluster

Cluster	Time period	Types of malaria	Observed cases	Expected cases	Relative risk (RR)	No. of blocks	p value
1	April 2011–March 2016	PV	8665	1439.44	7.42	24	< 0.001
		PF	7611	482.7	24.96		
2	April 2012–November 2016	PV	9184	2945.3	3.54	34	< 0.001
		PF	1455	1053.55	1.41		
3	June 2017–December 2017	PV	2025	81.67	25.69	7	< 0.001
		PF	44	29.22	1.51		
4	August 2017–October 2017	PV	1080	276.38	3.97	62	< 0.001
		PF	NA	NA	NA		
5	February 2018–November 2018	PV	337	22.79	14.87	2	< 0.001
		PF	9	8.15	1.1		
6	June 2013–July 2013	PV	NA	NA	NA	1	< 0.001
		PF	94	0.8	118.7		
7	January 2020–March 2020	PV	NA	NA	NA	1	< 0.001
		PF	95	1.57	60.89		
8	July 2011–September 2011	PV	NA	NA	NA	1	< 0.001
		PF	63	1.08	58.29		
9	June 2011–August 2013	PV	220	56.28	3.92	1	< 0.001
		PF	NA	NA	NA		

**Fig. 5:**
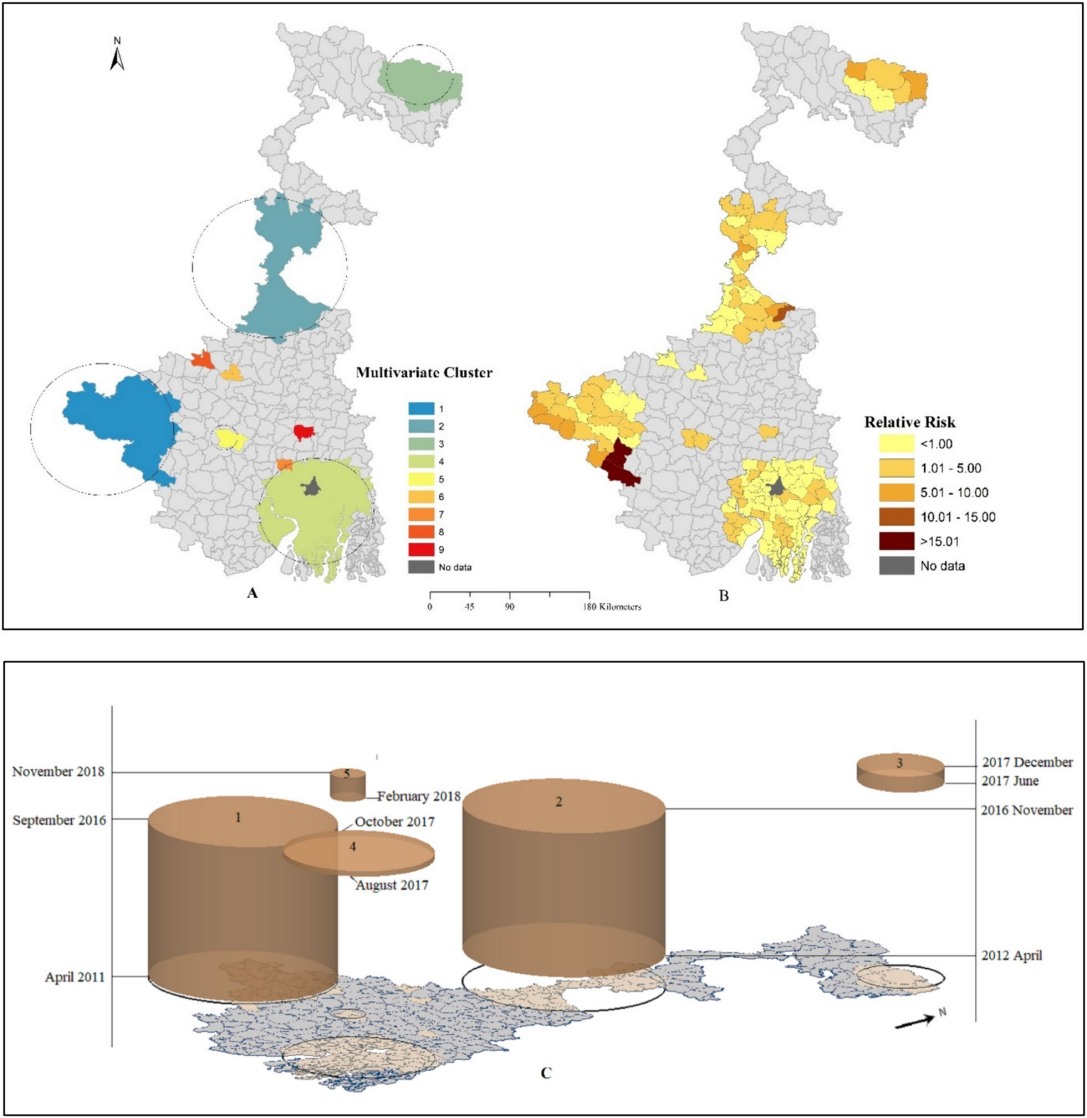
**A** Multivariate space–time cluster, and **B** Statistically significant relative risk. **C** 3D visualization of multivariate space–time cluster

In comparison with both results, there is the presence of the same clusters in a multivariate analysis that was also detected in univariate space–time *P. vivax* and *P. falciparum* analysis. Multivariate clusters 2, 3, and 4 are the same group of clusters that contain the same blocks in univariate *P. vivax* clusters 3, 2 and 4, whereas multivariate cluster 1 contains a portion of *P. falciparum* cluster 1. Figure 5A represents the multivariate cluster, which represents the co-occurrence of *P. vivax* and *P. falciparum* cases from April 2011 to March 2021. Figure 5B depicts the statistically significant relative risk of multivariate clusters of each block from the space–time cluster analysis. In multivariate analysis, the co-occurrence of *P. vivax* and *P. falciparum* collectively impacts 133 blocks. Among these, 68 blocks reported a relative risk of less than 1, while the remaining 65 blocks detected a relative risk of more than 1. After all, multivariate space–time scan statistics helped to identify the co-occurrence of both *P. vivax* and *P. falciparum*. In contrast, the multivariate study highlights the regions significantly affected by both malaria cases. Further studies are required to understand the complex spatial variation of malaria parasites.

## Discussion

Using ten years of retrospective surveillance data, this study demonstrated seasonal variability and significant space–time clusters of *P. vivax* and *P. falciparum* malaria at the block level in West Bengal during April 2011 to March 2021. Throughout the study period, seasonal occurrence of both malaria cases maximized during monsoon and post-monsoon, which follow a seasonal oscillating pattern, signify a strong relation with monsoon climate. Though for both of the cases seasonal trends show a gradual decline over the year, which signify successful control measures, improved healthcare, or natural fluctuations. During monsoon season, favourable environmental conditions and vector and human behaviour accelerate the breeding and growth of malaria parasites as well as transmission. Even malaria parasites can remain dormant for a long time until they get favourable conditions. As in West Bengal peak transmission seasons coincide with specific months monsoon and post monsoon, therefore, focused interventions are required, including monitoring and distribution of LLINs, ITN, vector control strategies with chemical and biological, community participation, environmental management, garbage cleaning, and awareness campaign should be strengthened to interrupt local transmission [7]. West Bengal’s diverse ecosystems, climate, and population density impact malaria transmission. The industrial/mining belt in West Bengal along with Jharkhand and Orissa has historically been a hotspot for malaria [30]. Monitoring local meteorological data and understanding vector behaviour are essential for accurate seasonal predictions and seasonal forecasting models can help predict malaria risk in advance [31].

With the help of retrospective univariate and multivariate space–time scan statistics, both analyses detected significant clusters of *P. vivax* and *P. falciparum* individually and its co-occurrence. From the analysis, non-random distribution of malaria cases have been found to create different sizes of clusters of boththrough the study area in different periods. From the analysis cluster 1 of *P. vivax* was detected in Jhargram district during 2011 to 2014. Here high tribal and marginalized population groups living in improvised condition [32] and have an interstate border with Jharkhand may cause the uplifted clusters. High-risk spatiotemporal clusters of *P. vivax* (cluster 3) was detected across cross border area in 34 blocks from Maldah, Murshidabad and Birbhum districts located at the central part of West Bengal along with Bangladesh and Jharkhand border during 2012 to 2016. Another high-risk spatiotemporal cluster of *P. vivax* (cluster 2) was found in northern part along with Bhutan and Assam border. Although, these areas are considering as porous border with Bangladesh which act as cross border illegal migration [33, 34]. Therefore, cross border population movement and sharing a long international border are likely to be a constant danger of influx of malaria cases from high-endemic to low-endemic areas [33–36]. An intriguing observation from the scan statistics perspective is the spatial shifting of *P. vivax* clusters. It is noteworthy that clusters 2, 4, 5, and 7 emerged in different geographical foci after 2017. This observation opens up opportunities for further exploration and investigation, which could lead to a better understanding of the underlying factors that drive the emergence and shifting of these clusters. The lack of spatial clusters of *P. vivax* after 2018 could be attributed to the successful control measures, such as the effects of LLINs or improved healthcare, which might have disrupted the natural transmission dynamics of malaria afterwards.

High-risk spatiotemporal cluster of *P. falciparum* was observed in the western part of West Bengal with Puruliya, Bankura, Jhargram districts covered by forest area. This cluster was also identified as the most prolonged time high-risk zone before 2017 and persisted over five years, contributing more than 85% *P. falciparum* cases. Dense forests, hills, perennial streams and high tribal and marginalized population groups living in improvised conditions [32, 37] potentially contribute to this situation. The risk of malaria transmission across these regions are quite high as local population living in the forest fringe and tribal areas which is physio-graphically mosquito-prone area. It appears that the concentration of *P. falciparum* in cluster 1 may be due to cross-border migration with high-endemic inter states, such as Odisha, Chhattisgarh, and Jharkhand [4, 32]. These states have reported a significant number of malaria cases caused by *P. falciparum*, which could explain the higher incidence of this particular strain in cluster 1. To gain a more comprehensive understanding of the situation, it would be helpful to investigate other factors such as the local climate, population density, and healthcare access in the region. However, prevalence of malaria cases decreased in Puruliya district from 2017 onwards which is possible with proper intervention and distribution of LLINs on time [38].

The present study also identified expansion in the geographical distribution of *P. falciparum* over *P. vivax* in the southern part of the Gangetic plain, especially after 2017. The emergence of *P. falciparum* clusters in the newer regions requires targeted interventions to disrupt further transmission. As the elimination process is progressing to be malaria-free by 2027 and to eliminate the disease by 2030, the road ahead is bumpy and beset with many challenges, including the emergence of a set of new asymptomatic or sub-microscopic malaria parasitaemia [39–42], the emergence of artemisinin and multidrug-resistant malaria [43, 44]. Likewise, eliminating malaria fever incidence and measuring transmission requires a robust monitoring system that facilitates early infection detection and quick, efficient action.

From the analysis distinct patterns were seen in the spatial variations between *P. vivax* and *P. falciparum* malaria, indicating that the biological features of the parasites varied and might have played a role in their transmission. The activity of vectors and the length of parasite incubation are influenced by climate, and these factors are associated with a higher risk of malaria [45]. *Plasmodium falciparum* needs a slightly higher temperature for parasite growth than *P. vivax*. As for both species, the minimal threshold temperature is around 18 and 15 °C, respectively; there is a greater chance that *P. falciparum* may spread to formerly colder locations due to global climate change [46]. Further investigation is required to fully understand the risk factors driving the geographic distribution and spatial shifting of *P. falciparum* throughout West Bengal.

Furthermore, the univariate study independently examines spatial and temporal variation and highlights the burden of *P. vivax* and *P. falciparum*. In contrast, the multivariate study highlights the regions significantly affected by both malaria species. However, the co-occurrence in four high-risk clusters signifies the transmission dynamics, though no clinical or microscopic co-infection has been recorded. Four multivariate clusters with *P. vivax* and *P. falciparum* were observed from April 2011 to November 2018. The coexistence of both malaria in the same geographical region within the same climatic condition indicates that seasonal factors may influence the co-occurrence, giving a call for vector control monitoring. Given the frequent coexistence of *P. vivax* and *P. falciparum* in India, prioritizing intervention actions becomes challenging due to potential variations in disease transmission between these species [9, 47]. As the objective of this is to identify the existence of space–time clusters and seasonal variability of malaria vectors, consequent studies are required to identify determining factors associated with this co-occurrence.

However, this study has a variety of limitations. First, for this study block level administrative unit has been taken but small-scale, such as village level unit, will help better understanding for disease outbreak and surveillance. Second important limitation is relative risk reported by each cluster. Reported relative risk for each cluster was detected underlying total population, which does not consider sociodemographic variation. In areas of low transmission all age group are vulnerable but adults develop more severe and multiple complications. In areas of high transmission children below 5 years, visitors and migratory labour are higher at risk. Pregnant women are less capable of coping with and clearing malaria infection, adversely affecting the unborn fetus. Therefore, adjusted rate based on age, sex and other socio-economic parameters might generate accurate relative risk. Third, though SaTScan is an important statistical tool for spatial and space time cluster analysis, challenging to determine the maximum window limit for spatial and temporal extension, as a result misdiagnosis of small and heterogenous clusters. Last, block level population data was taken from last census occurred in 2011. Using outdated demographic data is a critical issue effecting the findings of the research and a common limitation in many studies in developing countries. Despite having various limitations, this study is a first attempt to examine the seasonal variability and spatio-temporal cluster of *P. vivax* and *P. falciparum* malaria and its co-occurrence at the block level using 120 months longitudinal dataset. Identifying the covariate effects and transmission in the spatiotemporal context, ecological modelling like autoregressive study is required which will avenue the future target.

## Conclusion

Overall, seasonal trends from the time series decomposition analysis show a gradual decline for both *P. vivax* and *P. falciparum* cases in West Bengal. Whereas the space–time scan statistics identified high-risk blocks for *P. vivax* and *P. falciparum* malaria and its co-occurrence. Both malaria types exhibit significant spatiotemporal variations over the study area. The detected space–time clusters and the relative risk of each cluster were also visualized in both 2D and 3D to improve the understanding of the malaria epidemic through space and time analysis. Identifying emerging high-risk areas of *P. falciparum* malaria over the Gangetic belt indicates the need for more research for its spatial shifting. Moreover, to achieve the ambitious goal of becoming malaria-free by 2027 and eliminating the disease by 2030, these findings can play a significant role in prioritizing control programmes where disease prevention and control lending community support are much needed along with following up cross-border migration. Supplying LLINs and IRS, and the advent of ACT and application of more sensitive diagnostic tools, such as polymerase chain reaction (PCR) and loop-mediated isothermal amplification (LAMP), together with conventional methods to overcome the challenge of malaria elimination. Addressing the determinants of residual malaria transmission in the high-risk blocks within these varied clusters necessitates regional collaboration and strategic plans to combat malaria and future outbreak, which are critical steps towards overcoming the remaining hurdles in malaria eradication.

## Data Availability

All data analysed in this study was taken from the Ministry of Health and Family Welfare, Government of India.

## Abbreviations

GTS: Global technical strategy
APLMA: Asia Pacific Leaders Malaria Alliance
NFME: National Framework for Malaria Elimination
EPDT: Early case Detection and Prompt Treatment
NVBDCP: National Vector Borne Disease Control Programme
ITN: Insecticide-treated bed nets
LLIN: Long-lasting insecticidal nets
ACT: Artemisinin-based combination therapy
IRS: Indoor residual sprays
IVM: Integrated vector management
STSS: Space time scan statistics
RR: Relative risk
